# Supplementary material for: Normal manual straight ahead pointing in Complex Regional Pain Syndrome
Source: PLoS One. 2021 Dec 20;16(12):e0261614. doi: 10.1371/journal.pone.0261614 (PMC8687552; doi:10.1371/journal.pone.0261614)
Supplement: Attachment — Submitted filename: Response to Reviewers.docx [file pone.0261614.s001.docx]

PONE-D-21-14359
Normal manual straight ahead pointing in Complex Regional Pain Syndrome
PLOS ONE

Dear Dr. Vittersø,

Thank you for submitting your manuscript to PLOS ONE. After careful consideration, we feel that it has merit but does not fully meet PLOS ONE’s publication criteria as it currently stands. Therefore, we invite you to submit a revised version of the manuscript that addresses the points raised during the review process.

Before we can send this manuscript out for review, it needs to meet PLOS ONE's criteria for studies that are closely related to existing work. This study seems to address the same core hypothesis as the authors' 2021 Cortex paper, by again showing no evidence of a visuospatial bias in patients with CRPS. While the current study represents a new method of addressing this question, it does so without adequately discussing the previous work. For example, if these two manuscripts represent two methods addressing the same hypothesis, splitting it into two manuscripts does not remove the need for appropriate multiple-comparison testing.

In addition, given this manuscript's negative/weak results, it needs to discuss whether the sample size was adequate to address the question.

Please submit your revised manuscript by Jun 27 2021 11:59PM. If you will need more time than this to complete your revisions, please reply to this message or contact the journal office at [plosone@plos.org](mailto:plosone@plos.org). When you're ready to submit your revision, log on to <https://www.editorialmanager.com/pone/> and select the 'Submissions Needing Revision' folder to locate your manuscript file.

Please include the following items when submitting your revised manuscript:

- A rebuttal letter that responds to each point raised by the academic editor and reviewer(s). You should upload this letter as a separate file labeled 'Response to Reviewers'.
- A marked-up copy of your manuscript that highlights changes made to the original version. You should upload this as a separate file labeled 'Revised Manuscript with Track Changes'.
- An unmarked version of your revised paper without tracked changes. You should upload this as a separate file labeled 'Manuscript'.

If you would like to make changes to your financial disclosure, please include your updated statement in your cover letter. Guidelines for resubmitting your figure files are available below the reviewer comments at the end of this letter.

If applicable, we recommend that you deposit your laboratory protocols in protocols.io to enhance the reproducibility of your results. Protocols.io assigns your protocol its own identifier (DOI) so that it can be cited independently in the future. For instructions see: [http://journals.plos.org/plosone/s/submission-guidelines#loc-laboratory-protocols](https://eur01.safelinks.protection.outlook.com/?url=http%3A%2F%2Fjournals.plos.org%2Fplosone%2Fs%2Fsubmission-guidelines%23loc-laboratory-protocols&data=04%7C01%7Cadv22%40bath.ac.uk%7Cb83ede2a85194739aa5608d9190ab97a%7C377e3d224ea1422db0ad8fcc89406b9e%7C0%7C0%7C637568356118179567%7CUnknown%7CTWFpbGZsb3d8eyJWIjoiMC4wLjAwMDAiLCJQIjoiV2luMzIiLCJBTiI6Ik1haWwiLCJXVCI6Mn0%3D%7C1000&sdata=wQPlbvkz21p7RouiO9DWG%2B0KeJnWDcQni3Nlyfsb754%3D&reserved=0). Additionally, PLOS ONE offers an option for publishing peer-reviewed Lab Protocol articles, which describe protocols hosted on protocols.io. Read more information on sharing protocols at [https://plos.org/protocols?utm_medium=editorial-email&utm_source=authorletters&utm_campaign=protocols](https://eur01.safelinks.protection.outlook.com/?url=https%3A%2F%2Fplos.org%2Fprotocols%3Futm_medium%3Deditorial-email%26utm_source%3Dauthorletters%26utm_campaign%3Dprotocols&data=04%7C01%7Cadv22%40bath.ac.uk%7Cb83ede2a85194739aa5608d9190ab97a%7C377e3d224ea1422db0ad8fcc89406b9e%7C0%7C0%7C637568356118189528%7CUnknown%7CTWFpbGZsb3d8eyJWIjoiMC4wLjAwMDAiLCJQIjoiV2luMzIiLCJBTiI6Ik1haWwiLCJXVCI6Mn0%3D%7C1000&sdata=SYoqb2sAJQkREze56IfdsCoSsW2rXuSiZQqpf7W3Vcw%3D&reserved=0).

We look forward to receiving your revised manuscript.

Kind regards,

Benjamin A. Philip
Academic Editor
PLOS ONE

Journal Requirements:

When submitting your revision, we need you to address these additional requirements.

1. Please ensure that your manuscript meets PLOS ONE's style requirements, including those for file naming. The PLOS ONE style templates can be found at

<https://journals.plos.org/plosone/s/file?id=wjVg/PLOSOne_formatting_sample_main_body.pdf> and

[https://journals.plos.org/plosone/s/file?id=ba62/PLOSOne_formatting_sample_title_authors_affiliations.pdf](about:blank)

18^th^ of June 2021

PONE-D-21-14359 (resubmission)
Normal manual straight ahead pointing in Complex Regional Pain Syndrome

We would like to thank the editor for his helpful comments on our manuscript. We have revised it following his suggestions, and we believe that the paper is stronger as a result.

The editor’s comments are in bold, followed by our response. Where we have made significant changes to the manuscript, we have also included this altered text in red, below our response as indented text. The location of these changes in the text (version without tracked changes) is indicated in the square brackets. We numbered the editor’s comments, to make it easier to navigate the document.

Sincerely,

Dr. Axel D. Vittersø (Lead author)

Department of Psychology, University of Bath (UK)

Response to editor

**1. Before we can send this manuscript out for review, it needs to meet PLOS ONE's criteria for studies that are closely related to existing work. This study seems to address the same core hypothesis as the authors' 2021 Cortex paper, by again showing no evidence of a visuospatial bias in patients with CRPS. While the current study represents a new method of addressing this question, it does so without adequately discussing the previous work. For example, if these two manuscripts represent two methods addressing the same hypothesis, splitting it into two manuscripts does not remove the need for appropriate multiple-comparison testing.**

We thank the editor for pointing out this lack of clarity to us. In the process of addressing the editor’s comments, we have taken the opportunity to expand on and develop some of the ideas that were outlined in the original submission. We believe the manuscript is stronger because of these changes.

We appreciate that there are similarities between the submitted manuscript and our 2021 Cortex papers (i.e. Ten Brink et al., 2021; Vittersø et al., 2021), and the need to clarify their unique contributions to our understanding of neuropsychological changes in Complex Regional Pain Syndrome (CRPS).

Spatial representations are related to visuospatial attention, yet distinct. Visuospatial attention is concerned with the amount of attention that is devoted to a segment of space (e.g. left/right, near/far). In contrast, MSA depends on proprioceptive information and egocentric representations of space. The representation of space is typically considered distinct to the amount of attention that is given to a particular segment of space. Furthermore, most measures of visuospatial attention are allocentric by definition, such as line bisection, and are therefore more closely related to a different type of spatial information than MSA.

In Ten Brink et al. (2021, Cortex) we investigated a visuospatial attention bias. All tasks were concerned with visual attention, with several tasks involving eye tracking. In that study we were specifically interested in the contribution of body representations to visuospatial attention biases. For instance, we looked at if a bias would be specific to body related information, such as an image of a hand compared to a vegetable. There were no measures of spatial representations or of subjective body-midline in Ten Brink et al. (2021, Cortex) or in Vittersø et al. (2021, Cortex).

We have now added some more detail to our introduction and discussion to make it clear how the current manuscript relates to previous work.

[Introduction, p. 3]

Recently we showed that people with CRPS do not show a consistent visuospatial attention bias [8, 9]. However, some studies that have suggested biases in the representations of space (i.e. the mental knowledge or model of external space). For example, when asked to indicate when a visual target passes the point in front of the body midline in an otherwise darkened room (“visual straight ahead” judgements; VSA), people with CRPS have shown a bias away from the affected arm [10-14], a leftward bias [15] (i.e. pseudoneglect), or no bias [16, 17]. When performed in the dark, VSA is thought to reflect any lateral shifts representations of the body midline and of external space relative to one’s own position (i.e. egocentric spatial representations) [14, 18]. However, when performed in light conditions, the availability of other visual cues makes it possible to recruit allocentric representations (i.e. the representation of objects in space relative to each other). The observation of normal VSA judgments made by people with CRPS in light conditions [14] therefore suggest that only egocentric, and not allocentric, representations of space are biased.

We have also added some more detail to clarify how VSA and MSA are related, yet distinct.

[Introduction, p. 3]

Manual straight ahead (MSA) is related to VSA, and involves pointing straight in front of one’s perceived body midline without visual feedback of the pointing arm’s location (e.g. with the eyes closed). MSA has previously been used to quantify directional biases of the perceived body midline in an egocentric reference frame in healthy controls [e.g. 19, 20-23], and people with post-stroke neglect. Like VSA in dark conditions, MSA is thought to reflect egocentric representations of space [24]. However, unlike VSA, MSA can also provide insights into proprioceptive accuracy (i.e. in the pointing arm), and the ability to align the felt position of the arm with the perceived direction of straight ahead. Two studies found that people with CRPS were more variable than control participants when matching the position of their affected or unaffected arm to externally-defined targets [25, 26], indicating impaired proprioception. It could therefore be informative to use MSA to gain further insights into both spatial representations and limb proprioception, and also to evaluate whether there are also deficits in the way that arm proprioceptive information is combined with egocentric spatial representations.

We have added a more detailed consideration of our findings in the discussion, and how they relate to previous findings more broadly.

[Discussion, p. 16]

Our study adds to an emerging pattern that MSA has generally been reported as unbiased in CRPS, whereas the evidence is less consistent for the presence or absence of a VSA bias. Discrepancies in the spatial biases in CRPS could relate to the different reference frames (egocentric, allocentric), regions of space (peripersonal, extrapersonal), and/or qualities (goal-directed, defensive) of the space being tested [3]. For instance, VSA is thought to predominantly rely on egocentric reference frames [14, 18], whereas MSA also involves proprioceptive information [24]. The targets in VSA are typically presented at a distance from participants (e.g. 260 cm away in Verfaille et al. [27]), and thus are in extrapersonal/far space. In MSA, the “target” is a felt position and thus would be considered to be located in personal space and/or imaginary space. These task differences might have given rise to the finding that MSA is unbiased for people with CRPS [17, 27, 28], while some studies suggested that VSA is biased [10-15]. However, it should be noted that the findings of VSA bias in CRPS are not consistent, and that the largest study did not find any evidence of a bias [n = 53; 16].

[Discussion, p. 17]

Recent studies have showed that people with CRPS differ from pain-free controls in how they integrate bodily and visual information [44], and in how they update bodily and spatial representations when interacting with external objects [i.e. tools; 45]. It is possible that this altered integration and/or updating is related to the deficits that are observed on arm matching tasks for people with CRPS. Alternatively, given that our Bayesian analysis was inconclusive about whether the null could be supported, it could be that more subtle differences in MSA variability could be detected in studies involving more participants.

[Conclusions, p. 18]

In contrast to potential biases in VSA pointing [10-15], our study corroborates past research showing no directional bias in MSA pointing in CRPS [17, 27, 28]. This finding complements recent findings that show no visuospatial attention bias in people with CRPS [8, 9, 47] by showing that egocentric spatial representations are also unaffected. The inconsistent findings of spatial biases in CRPS could relate to the different reference frames, regions of space, and/or qualities of the space being tested [3]. We did not find any evidence of impaired proprioception for people with CRPS, which, although consistent with previous MSA findings [28], contrasts findings from position matching task [25, 26]. Therefore, reconciling these discrepant findings is needed to further our understanding of neurocognitive changes in CRPS.

All the participants in the present manuscript also took part in a study we have previously published (Vittersø et al. 2021, Cortex). This study was focused on characterising the process of sensorimotor adaptation (i.e. the change in response to perturbation, rather than stable bias per se). Although there was some overlap, the participants in Ten Brink et al. (2021, Cortex) were different to the ones in the current manuscript. Participants who took part in both studies typically did so on separate sessions. More importantly, the current study and that of Ten Brink et al. (2021, Cortex) had their own research aims, research protocol, funding, ethical approval, and preregistrations. We therefore do not consider this an example of multiple-comparison testing.

We have added some detail to our manuscript to make it clear how the current manuscript differs from Vittersø et al. (2021, Cortex), and that the participants in the two studies were the same.

[Method, p. 5]

After completing the MSA task, all participants also took part in a study that aimed to characterise the process of sensorimotor adaptation [23]. The current study is reported separately because it was focused on assessing the presence of any systematic bias in MSA for people with CRPS, rather than the difference in the transfer of sensorimotor prism adaptation after-effects to spatial representations [e.g. 24].

**2. In addition, given this manuscript’s negative/weak results, it needs to discuss whether the sample size was adequate to address the question.**

We thank the editor for raising this point.

We would like to clarify that moderate evidence for the null (i.e. BF_10_ < 1/3) should not be confused with a *p*-value above .05 (Wagenmakers et al., 2018, Psychonomic bulletin & review), as only the former provides evidence for the null hypothesis. We found moderate evidence of no effect (i.e. BF_10_ < 1/3; Wagenmakers et al., 2018) for our main MSA outcome measures, which suggests that our study was sufficiently powered to detect a “significant effect” in Bayesian terms. A moderate effect is within the level of certainty that would typically be accepted for a Bayesian analysis (Wagenmakers et al., 2018) and what we preregistered as our inference criteria (<https://osf.io/6jpfg/>).

To add clarity, we have added an outline of our statistical approach to our method section.

[Method, p. 13]

We conducted frequentist and Bayesian repeated measures analyses of variance (ANOVAs) to address our hypotheses related to MSA errors and MSA variability. We calculated the mean MSA error and its variability (i.e. SD) from the 10 trials for each participant and for each hand. To address the hypothesis related to the presence of a directional bias in spatial representations, we compared Groups (CRPS, Controls), and Side of Body (affected/non-dominant, non-affected/dominant) on MSA errors. To address the question related to proprioceptive precision, we compared Groups and Side of Body on MSA variability. To see if spatial biases were related to CRPS symptoms and/or disability, we performed correlational analyses of the relationship between MSA, clinical data, and questionnaire measures.

We considered a p-value < 0.05 as statistically significant for frequentist analyses. For Bayesian analyses we report Bayes Factors (BF) expressed in favour of the alternative hypothesis (i.e. BF10), analysed using an uniformed uniform prior. We considered a BF10 of 1-3 as inconclusive/anecdotal evidence for the alternative hypothesis, 3-10 moderate evidence, and 10-30 strong evidence [29]. In contrast, we considered a BF10 of 1/3-1 as inconclusive/anecdotal evidence for the null hypothesis, 1/10-1/3 moderate evidence, and 1/30-1/10 strong evidence.

For the MSA variability our results were inconclusive (i.e. 1/3 < BF_10_ < 3), and we agree that our study might have been underpowered to detect any differences for this measure. We have added a consideration of whether the sample size was adequate to address the MSA variability.

[Discussion, p. 16]

We did not find evidence of any differences in MSA variability when comparing between groups or the arm used, consistent with previous research [28]. However, our Bayesian analysis did not provide evidence in favour of no difference.

[Discussion, p. 15]

Our finding of no difference between the variability of MSA errors of people with CRPS and controls contrasts with reports of bilateral proprioceptive deficits in CRPS on arm position matching tasks [25, 26]. These discrepancies could be due to the reference frames required. MSA is presumed to rely on an egocentric reference frame [24], whereas matching arm position to an external target depends on a combination of egocentric and allocentric reference frames [43]. Recent studies have showed that people with CRPS differ from pain-free controls in how they integrate bodily and visual information [44], and in how they update bodily and spatial representations when interacting with external objects [i.e. tools; 45]. It is possible that this altered integration and/or updating is related to the deficits that are observed on arm matching tasks for people with CRPS. Alternatively, given that our Bayesian analysis was inconclusive about whether the null could be supported, it could be that more subtle differences in MSA variability could be detected in studies involving more participants.

The number of people with CRPS in our study was larger or the same as previous studies of MSA in this population (n = 1; Jacquin-Courtois et al., 2017, Cortex; Christophe et al., 2016, Annals of physical and rehabilitation medicine; n = 7; Christophe et al., 2016, Neural Plasticity; n = 17; Verfaille et al., 2020, Pain). Only one study had a larger sample size (n = 20; Kolb et al., 2012, Pain). We make explicit reference to the sample size of each of these studies in the introduction (p. 3-4). We have added some more detail to the discussion about how our sample size compares to past MSA studies of people with CRPS.

[Discussion, p. 15]

We found moderate evidence for no difference between people with CRPS and matched controls on directional MSA endpoint errors. This finding is consistent with previous MSA research [17, 27, 28], although our study used a larger sample than most previous studies [10, 11, 17].

We have also added a consideration of our sample size and how it compares to the most recent MSA study.

[Discussion, p. 16]

The only other study to test the equivalence of MSA errors of people with CRPS and pain-free controls was Verfaille and her colleagues [27]. They found inconclusive/anecdotal evidence in favour of no difference when coded relative to the CRPS-affected side of space. Their sample was the same size as ours (i.e. 17 people with CRPS). Although largely comparable, the subtle differences in findings could be due to methodological differences. For instance, we recorded endpoint errors using a motion capture system sensitive to ±1.4 mm. In the study by Verfaille and her colleagues [27], the experimenter visually inspected endpoint errors against a protractor that was precise to 1°. This difference could have been sufficient to enable our study to detect evidence for the null hypothesis, whereas the findings of Verfaille and her colleagues were inconclusive/anecdotal. It is, however, noteworthy that our findings are largely compatible considering that our study involved people with more chronic CRPS (M duration = 72.65, SD = 41.62) compared to the sample tested by Verfaille and her colleagues (M duration = 13.09, SD = 9.36).
