# Supplementary material for: Normal manual straight ahead pointing in Complex Regional Pain Syndrome
Source: PLoS One. 2021 Dec 20;16(12):e0261614. doi: 10.1371/journal.pone.0261614 (PMC8687552; doi:10.1371/journal.pone.0261614)
Supplement: Attachment — Submitted filename: Response to reviewers Rev1.docx [file pone.0261614.s002.docx]

PONE-D-21-14359R1

Normal manual straight ahead pointing in Complex Regional Pain Syndrome

PLOS ONE

Dear Dr. Vittersø,

Thank you for submitting your manuscript to PLOS ONE. After careful consideration, we feel that it has merit but does not fully meet PLOS ONE’s publication criteria as it currently stands. Therefore, we invite you to submit a revised version of the manuscript that addresses the points raised during the review process.

Please make sure to address - by revisions or explanation - the major concerns of Reviewer #1.

Please submit your revised manuscript by Nov 11 2021 11:59PM. If you will need more time than this to complete your revisions, please reply to this message or contact the journal office at plosone@plos.org. When you're ready to submit your revision, log on to https://www.editorialmanager.com/pone/ and select the 'Submissions Needing Revision' folder to locate your manuscript file.

Please include the following items when submitting your revised manuscript:

·	A rebuttal letter that responds to each point raised by the academic editor and reviewer(s). You should upload this letter as a separate file labeled 'Response to Reviewers'.

·	A marked-up copy of your manuscript that highlights changes made to the original version. You should upload this as a separate file labeled 'Revised Manuscript with Track Changes'.

·	An unmarked version of your revised paper without tracked changes. You should upload this as a separate file labeled 'Manuscript'.

If you would like to make changes to your financial disclosure, please include your updated statement in your cover letter. Guidelines for resubmitting your figure files are available below the reviewer comments at the end of this letter.

If applicable, we recommend that you deposit your laboratory protocols in protocols.io to enhance the reproducibility of your results. Protocols.io assigns your protocol its own identifier (DOI) so that it can be cited independently in the future. For instructions see: https://journals.plos.org/plosone/s/submission-guidelines#loc-laboratory-protocols. Additionally, PLOS ONE offers an option for publishing peer-reviewed Lab Protocol articles, which describe protocols hosted on protocols.io. Read more information on sharing protocols at https://plos.org/protocols?utm_medium=editorial-email&utm_source=authorletters&utm_campaign=protocols.

We look forward to receiving your revised manuscript.

Kind regards,

Benjamin A. Philip

Academic Editor

PLOS ONE

2^nd^ of November 2021

PONE-D-21-14359R1 (resubmission)
Normal manual straight ahead pointing in Complex Regional Pain Syndrome

We would like to thank the reviewers for their helpful comments on our manuscript. We have revised it following their suggestions, and we believe that the paper is stronger as a result.

The reviewers’ comments are in bold, followed by our response. Where we have made significant changes to the manuscript, we have also included this altered text in red, below our response as indented text. The location of these changes in the text (version without tracked changes) is indicated in the square brackets. We numbered the editor’s comments, to make it easier to navigate the document.

Sincerely,

Dr. Axel D. Vittersø (Lead author)

Department of Psychology, University of Bath (UK)

Reviewer comments

**Reviewer #1:**

**Review Vitterso et al. Normal manual straight ahead pointing in Complex Regional Pain Syndrome.**

**Dear authors,**

**sorry that this took a while to do this review.**

We thank the reviewer for finding the time to review our manuscript and appreciate that they have many other demands on their time.

**1. In the paper you present a case series of 17 patients with CRPS in contrast to control subjects that were age and gender matched.**

**There were no findings that indicate a difference between the 2 groups in the manual straightahead while there are ambiguous evidences for deviations towards the unaffected side („neglect- like“) from other studies.**

**You present an established paradigm and adequately point out the difference between the different reference frames and therefor the reasoning for choosing the presented paradigm over VSM. I wish we could compare the results of VSM to MSA in the same subjects to compare the methods which would give us more insights on the different mechanisms of the two tasks and the involved pathophysiologic basis. But anyways from most of the studies there is no effect in VSM as there is no effect in MSA. Yet, your paradigm is not very innovative from my point of view but the statistical approach indeed is (to be honest: I think analysing all the negative studies on pointing tasks in a similar way as you did might lead to comparable results).**

We are glad that the reviewer recognises the value of our statistical approach and the established paradigm that we used.

Two previous studies have investigated both VSA and MSA in CRPS. Chrisophe et al. (2016, Neural Plasticity) compared people with left upper-limb CRPS to those with a right upper-limb CRPS on MSA and VSA. They did not find any difference between groups. Similarly, Verfaille et al. (2020, Pain) compared people with CRPS to pain-free controls on VSA and MSA. They did not find any significant deviations from zero in VSA or MSA for either group, or any between-group differences.

**2. I do not fully agree with the notion that involving the sensory system in terms of proprioception in a pointing task will improve the egocentricness of this task as sensory symptoms and muscular changes affecting proprioception are part of the diagnosis of CRPS and impaired hand size estimation (Petz et al. 2011) is also a symptom of CRPS as well as loss of proprioceptive accuracy in positioning tasks (Lewis 2010, which you also cite but maybe interpret it in a more „centralistic“ way than I do), so I would argue against any positive finding of the MSA- pointing showing a between groups effect as equivalent of egocentric representation which is not the case in this study so may be not a problem but maybe you should weigh the effects of sensory symptoms in the reasoning of choosing your paradigm and reconsider the CRPS- unbiased egocentricness of it always keeping in mind that we are searching for central representation mechanisms and not peripheral effects of CRPS. This is in some way addressed in lines 139 ff. but in the paragraphs before concepts get a bit mixed up.**

The reviewer is correct in pointing out that many people with CRPS experience their affected area as larger (i.e. distorted body representations) and that proprioception can be impaired, although neither of these are part of the diagnostic criteria for CRPS (Harden et al., 2010, Pain). That being said, most of our participants reported that their affected area felt larger than its physical size, and self-reported body representation distortion correlated with CRPS severity. We also appreciate that proprioceptive deficits in CRPS can have a peripheral origin. However, as the reviewer alludes to, the finding of bilateral deficits in unilateral CRPS (Lewis et al., 2010, Pain) suggests that the deficits might also have a central component. We agree with the reviewer that it is important to look at both central and peripheral mechanisms in CRPS, and any interaction between central and peripheral mechanisms. The latter might be most relevant to the current manuscript.

We do not think that proprioceptive information makes the MSA task more egocentric, but rather that participants will have more sensory information available to them than during VSA. For instance, the type of sensory information available during pointing has been found to influence pointing accuracy for people with CRPS (Verfaille et al., 2020, Pain).

We have added some detail to clarify our reasoning in the introduction.

[Introduction, p. 4]

Furthermore, a recent study of people with CRPS found that their pointing accuracy depended on the type of sensory information available (visual, proprioceptive, or visual-proprioceptive) [27]. It could therefore be informative to use MSA to gain further insights into both spatial representations and limb proprioception, and to evaluate whether there are also deficits in the way that arm proprioceptive information is combined with egocentric spatial representations.

**3. Though not really large, the group of patients is larger than in comparison to other studies with manual straightahead pointing tasks but on the other hand the effects one would expect from those studies are not that large that I can imagine that those studies went into power calculation for the study presented here.**

**The choosing of sample sizes for pragmatic reasons is really honest but does not seem very scientific to me, which is my point of biggest concern and which limits the impact of the findings a lot (see also next paragraph). Anyways you present not only the absence of evidence for a difference between groups (which would be most likely of doubt because of the number of subjects) but the actual evidence for absence of difference between groups. So leaving the generally sparse (and certainly underpowered from a statistical point of view) number of subjects out of the scope, what seems like a statistical trick at first sight, might indeed add some crucial point to the scientific debate (if maybe it is only to stop doing pointing tasks with our patients).**

We thank the reviewer for recognising the value that our study adds to the scientific debate. We agree that null-results are important to consider, and to address appropriately (e.g. by providing evidence for the null-hypothesis through Bayesian analysis).

As the reviewer points out, our sample size is larger than or comparable to previous studies of MSA in people with CRPS (n = 1: Jacquin-Courtois et al., 2017, Cortex; Chrisophe et al., 2016, Annals of physical and rehabilitation medicine; N = 7: Chrisotphe et al., 2016, Neural Plasticity; n = 17: Verfaille et al., 2020, Pain). However, we disagree with the reviewer that a pragmatic approach to determining sample size is inherently unscientific (e.g. see Lakens, 2021, PsyArXiv for a discussion on how to improve the justification of sample size). All studies will be constrained by time and resources to a certain degree, particularly in the field of neuropsychology. In line with the open science movement, we have been transparent and honest about our approach and how it was shaped by the constraints that we were under. As the reviewer points out, our sample was sufficient to detect a significant effect in Bayesian terms. That being said, we acknowledge the limitation of having a sample of 17 patients, particularly when it comes to the correlational analyses. We have therefore made sure that all the correlational analyses are referred to as exploratory.

We have added a consideration of our sample size to the limitation section that we have added to our discussion.

[Discussion, p. 18]

Our study is not without limitations. The sample size meant that our study was only powered to reliably detect large effects, despite having more participants than most previous studies [10, 11, 17] of MSA in CRPS. Although not an aim of the study, this limitation meant that we were not able to explore the direct contribution of factors such as CRPS classification (i.e. not otherwise specified, clinical criteria, research criteria), the presence/absence of painful comorbid conditions, or handedness. The limited power also means that our exploratory correlation analyses should be interpreted with a degree of caution.

We also calculated what effect size our study was powered to reliably detect. We have added a description of this calculation in our method section.

[Method, p. 5]

The final sample size meant that the study was able to reliably detect a large effect ƞ_p_^2^ ≥ .21, with an alpha of .05, and 80 % power.

**4. I personally think that scientific studies for CRPS should not include subjects with the diagnosis CRPS NOS but only CRPS –patients at least fulfilling clinical criteria. I would be interested in the results of the study when leaving CRPS NOS- patients out of the statistical consideration. It might even improve the results due to loss of variance or due to loss of power lower the effect size.**

**I would also not include patients with other pain disorders such as FMS, knee damage or migraine in CRPS- studies.**

We agree that studies into CRPS would ideally be selective and, for instance, only include people who meet the clinical/research criteria and who have no comorbid pain diagnoses. However, the reality of conducting clinical research is far from ideal, and one must consider what is feasible. In a survey conducted by our group that included 478 people with CRPS (Ten Brink et al., 2020, *Pain*), on average people with this condition reported at least one other pain-related diagnosis, including fibromyalgia (18% of the group), migraine (11% of the group), and low back pain (26% of the group). As CRPS is relatively rare, we would not have been able to recruit a large enough sample for the present study size to draw meaningful conclusions if we were to exclude people with comorbid conditions in addition to those with CRPS NOS. Furthermore, excluding people with comorbid conditions would lead to a sample that is not representative of people with CRPS. In all cases, the CRPS was the primary concern of the participants in terms of pain conditions (that is, the condition that bothered them the most).

Bearing in mind the concerns that the reviewer raised in their previous point about our sample size (reviewer comment #3), we find it difficult to justify the removal of participants with CRPS NOS (n = 2) and/or other painful conditions (n = 7). Instead, we have created two new figures (Rev1-fig 1, Rev1-fig 2) to facilitate visual exploration of the data. These figures are only intended for review purposes and have not been included in the manuscript.

Endpoint errors from MSA pointing split by CRPS classification (NOS, clinical criteria, research criteria) are depicted in the figure below (Rev1-fig 1). Visual inspection of the data suggested that there were no clear patterns that emerged that depended on the CRPS classification (NOS, clinical criteria, research criteria) or CRPS severity for endpoint errors during MSA pointing, for either Hand (affected, non-affected).

**Rev1-fig 1**. Mean endpoint errors in degrees are presented for participants with CRPS, split by Side of Body (affected [A], non-affected [B]). People with CRPS are grouped by their clinical classification (NOS [green], clinical criteria [blue], research criteria [red]) and ordered by their CRPS severity score (y axis) within each clinical classification. A negative score on the x-axis indicates errors made towards the affected side.

Nonetheless, we did perform an analysis in case the reviewers would find it informative, and this showed that the direction of the results remained the same after removing the two people with CRPS NOS. That is, there was strong evidence for a difference between the affected/non-dominant and the non-affected/dominant hand (BF10 = 1150.51). There was moderate evidence of no difference between groups (BF10 = 0.29) and for no interaction between Group and Body side (BF10 = 0.07).

We have added a sentence to the results section to describe the consideration of how CRPS classifications might have influenced our results.

[Results, p. 14]

Furthermore, when we visually inspected the data split by different CRPS classifications (i.e. NOS, clinical criteria, research criteria), we did not see any indication that these results were shaped by the inclusion of people in these different diagnostic categories.

Next, we visually explored endpoint errors from MSA pointing, split by the presence/absence of comorbid painful conditions (Rev1-fig 2), such as fibromyalgia, migraine, and knee damage. There were no clear group differences for the affected hand (no comorbidities *M* = -5.52°, SD = 9.59; comorbid pain *M* = -3.72, SD = 8.68). For the non-affected hand, there was a tendency for people without any comorbid pain conditions (*M* = 5.42, SD = 6.01) to make larger endpoint errors than those with a comorbid pain condition (*M* = 0.56, SD = 4.28).

**Rev1-fig 2**. Mean endpoint errors in degrees are presented for participants with CRPS, split by Side of Body (affected [A], non-affected [B]). People with CRPS are grouped by their comorbidities (no painful commodities/NA [grey; n = 10], painful comorbidities [red; n = 7]). A negative score on the x-axis indicates errors made towards the affected side.

Our results did not change when we re-analysed the data after excluding people with CRPS who also had comorbid pain conditions. That is, in a 2x2 ANOVA (Group [CRPS without comorbid pain, controls], Side of Body [affected/non-dominant, non-affected/dominant]) there was still no difference between people with CRPS (without comorbid pain; n = 10) and controls on MSA endpoint errors, *F*(1,26) = 0.20, *p* = .657, BF_10_ = 0.3144, or any interaction with Side of Body, *F*(1,26) = 0.65, *p* = .429, BF_10_ = 0.503. We recognise that we had limited power to detect differences with this diminished sample size, however the Bayes Factor for the main effect indicated evidence for no difference (whereas the Bayes Factor for the interaction term was inconclusive). Overall, our results do not appear to change when we exclude people with comorbid pain conditions. We have added some detail to our results section to clarify this point.

[Results, p. 14]

The results were broadly similar when we re-expressed endpoint errors in terms of left (negative) and right (positive), when clinical measures were included as covariates, and when we excluded participants with comorbid pain conditions.

**5. Did you test for the effect of handedness in your statistical model and the interaction term of affected * dominance? What we know from other studies is that people tend to bisect a room more on the left side so I think that we need to account for the amount of pseudoneglect, which is more pronounced in right handed subjects than in left handed subjects as baseline correction of the statistical model or try to explore the interaction terms.**

We agree with the reviewer that it is important to consider the influence of handedness, for instance to account for differences in pseudoneglect. The potential effect of handedness was controlled for at the level of the study design by matching the control group for handedness. Only three of the 17 patients were lefthanded, so we do not have sufficient data to gain any meaningful insights into the influence of handedness on MSA pointing. In line with your earlier point (reviewer comment #3), we do not consider it appropriate to perform statistical analyses on so few data points. That being said, there is no reason the believe that the effect of handedness on MSA would differ between people with CRPS and controls, and thus it should not have influenced our main findings.

The results were also broadly similar when we expressed endpoint errors as left (negative) to right (positive), instead of affected/non-dominant (negative) and non-affected/dominant (positive), as described in line 226. Therefore, we did not find any evidence to suggest that the amount of pseudoneglect differed between groups.

We have added some detail to our discussion about the effect of handedness.

[Discussion, p. 18]

Our study is not without limitations. The sample size meant that our study was only powered to reliably detect large effects, despite having more participants than most previous studies [10, 11, 17] of MSA in CRPS. Although not an aim of the study, this limitation meant that we were not able to explore the direct contribution of factors such as CRPS classification (i.e. not otherwise specified, clinical criteria, research criteria), the presence/absence of painful comorbid conditions, or handedness.

**6. What are your thoughts about the negative significant correlation between the error of the affected side and the error of the affected side? Maybe they can elaborate on that in your discussion a bit more.**

We agree with the reviewer that this is an interesting point to consider. To untangle this correlation, we plotted the data (Rev1-fig 3A). The plot reveals that one outlier exerts quite a large influence on the correlation. The *r* value drops to -.68 after removing this individual. Hence, we must be cautious when interpreting this exploratory correlation (see response to reviewer comment #3).

**Rev1-fig 3**. Correlation between endpoint errors from MSA pointing for the affected/non-dominant hand (x-axis) and the non-affected/dominant hand (y-axis) with a trendline fitted. Correlations are depicted for people with CRPS (A) and control participants (B). Dots indicate mean values for each participant. A negative value indicates errors made towards the affected/non-dominant side.

We also considered what this association looked like for control participants (Rev1-fig 3B). The correlation between endpoint errors for the non-dominant and the dominant hand showed a similar trend to that observed between the non-affected and the affected hand for people with CRPS. For controls, there were no clear outliers and a somewhat weaker correlation that was non-significant (*r* = -.41, *p* = .091). We cannot make any between group comparisons on the basis of these correlations (e.g. Makin & de Xivry, 2019, eLife), which is more appropriately addressed by an ANOVA. Our original analysis included this comparison. Importantly, there was moderate evidence of no interaction between Group and Side of Body, so there is no reason to believe that this pattern was different between people with CRPS and controls.

Caveats aside, it looks like this correlation could be attributed to individual participants displaying similar biomechanical idiosyncrasies when pointing with each of their hands. Most participants tended to “undershoot” with each hand, resulting in a negative endpoint error with their affected hand and a positive endpoint error with their affected hand. Three people with CRPS showed the opposite pattern, “overshooting” with each hand. In our experience, such mirror-symmetrical patterns are not unusual.

We have added some detail about these findings in our results section.

[Results, p. 15]

MSA errors for the affected hand negatively correlated with MSA errors for the non-affected hand, reflecting that individual participants showed similar biomechanical reach behaviour for each hand (i.e. either undershooting or overshooting with each hand). A similar correlation was found for the control participants (*r* = -.41, *p* = .091), albeit non-significant.

**7. Concerning line 240 ff. : I think that we learned a lot about the influence of anxiety and kinesiophobia in CRPS in the recent years and their interrelation with peripersonal space and extrapersonal allodynia. Maybe we need to consider not only allocentric/egocentric reference frame but also the nearness of the task target to the peripersonal space. You addressed that in your conclusions but it might be a crucial point here.**

We agree with the reviewer that understanding how peripersonal space representations might be altered for people with CRPS is an interesting avenue for research. In fact, we have recently published a paper looking at the updating of peripersonal space representations in CRPS (Vittersø et al., 2020, Pain) that we refer to in line 309. In the present study, however, there was no measure of peripersonal space representations or any stimulation that varied in its distance to participants’ peripersonal space. Therefore, we consider an in-depth debate about peripersonal space representations in CRPS to be beyond the scope of the current manuscript, despite our shared interest in the topic.

We included a measure of fear of movement in our study, the Tampa Scale of Kinesiophobia (Kori, 1990, Pain Management). We did not find any association between fear of movement and endpoint errors during MSA pointing. Therefore, although fear of movement might play an important role in peripersonal space representations, we do not find any evidence to suggest that it was related to MSA pointing.

We have also had participants in previous studies who experienced “extrapersonal allodynia”, or conflict allodynia (McCabe & Blake, 2008, Rheumatology), and agree that their presentations is fascinating. None of the participants in the current study reported experiencing conflict allodynia, so we cannot make any inference about how MSA might be altered for this subgroup of people with CRPS.

**8. So in general I think this is a interesting footnote to the scientific debate rather than a very innovative fully grown paper. My greatest concern is the selection of subjects for your study and the number of subjects in your study which can not be improved post hoc but maybe should be addressed in a „limitations“- section.**

We have now added a limitations section to our discussion.

[Discussion, p. 18]

Our study is not without limitations. The sample size meant that our study was only powered to reliably detect large effects, despite having more participants than most previous studies [10, 11, 17] of MSA in CRPS. Although not an aim of the study, this limitation meant that we were not able to explore the direct contribution of factors such as CRPS classification (i.e. not otherwise specified, clinical criteria, research criteria), the presence/absence of painful comorbid conditions, or handedness. The limited power also means that our exploratory correlation analyses should be interpreted with a degree of caution.

**Minor comments:**

**Line 40-41 this sentence is a bit unclear, what is meant by „and according to the arm used“, I think this refers to „We compared endpoint errors“, but the sentence is a bit odd.**

**Lines 43 and 44 Groups is spelled with a capital G.**

**Line 48 „This study is consistent …“**

**Line 58 „although see“ --> rest of the sentence is missing**

**Line 66 „VSA is thought to reflect any lateral shifts of…“ --> missing word**

**Line 106 CRSP --> CRPS**

**Line 133 Groups and Side of Body spelled with capitals**

**Line 135 „we would expect to see a greater“ (the „a“ is a word to much)**

**Table 1 migraines --> migraine**

**Line 185 questionnaires measures („s“ or „measures“ to much)**

**Line 229 Groups and Side of Body spelled with capitals**

**Line 246 and 247 Groups and Side of Body spelled with capitals**

**Line 251 Groups and Side of Body spelled with capitals**

Thank you for bringing our attention to these typos. We have now corrected them in the text. The use of capital letters when spelling Group and Side of Body is deliberate, as they refer to factors in our analysis.

**Reviewer #2:**

**The authors have now addressed all concerns sufficiently and I now suggest to accept the manuscript as it stands now.**

We thank the reviewer for their positive appraisal of our work, and for reviewing the manuscript (again).

Please note that the data for the manuscript is openly available on the Open Science Framework (<https://osf.io/t9j52/>), as we refer to in line 213.
